# Association of genetic polymorphisms in interferon-γ, interleukin-6 and transforming growth factor-β1 gene with oral lichen planus susceptibility

**DOI:** 10.1186/s12903-016-0277-x

**Published:** 2016-08-20

**Authors:** Maha Ali M. Al-Mohaya, Lubna Al-Otaibi, Fahad Al-Harthi, Ebtissam Al Bakr, Misbahul Arfin, Abdulrahman Al-Asmari

**Affiliations:** 1 Department of Dentistry, Prince Sultan Military Medical City, Riyadh, Saudi Arabia; 2 Department of Dermatology, Prince Sultan Military Medical City, Riyadh, Saudi Arabia; 3 Research Center, Prince Sultan Military Medical City, P. O. Box 7897, Riyadh, 11159 Saudi Arabia

**Keywords:** Oral lichen planus, Interferon-γ, Interleukin -6, Transforming growth factor -β1, Polymorphism, Saudis

## Abstract

**Background:**

Oral lichen planus (OLP) is a premalignant mucocutaneous disease in which genetic factors and immune responses play a major role. Cytokines play an important role in the pathogenesis and disease progression of OLP. The aim of this study was to investigate the impact of gene polymorphisms of T helper cell subtype Th1 and Th2 cytokines, interferon-gamma (IFN-γ), interleukin-6 (IL-6) and transforming growth factor (TGF)-β1 on OLP susceptibility in a Saudi cohort.

**Methods:**

Forty two unrelated patients with OLP and 195 healthy controls were genotyped for IFN-γ (874A/T), IL-6 (174G/C) and TGF-β1 (509C/T) polymorphisms.

**Results:**

The frequency of genotype AT of IFN-γ (874A/T) was significantly higher while genotype AA was lower in OLP patients as compared to controls (*P* < 0.05). The frequency of T containing genotypes (AT + TT) was also higher in OLP patients as compared to that in controls (*P* = 0.003). The frequencies of allele T was higher while that of allele A lower in patients than the controls however the difference was not statistically significant (*P* = 0.07). There was no significant difference in the frequencies of alleles and genotypes of IL-6 (174G/C) and TGF-β1 (509C/T) polymorphisms between patient and control groups. These results indicated that genotype AT of IFN-γ (874A/T) polymorphism is associated with OLP risk and genotype AA is protective to OLP. On the other hand the polymorphisms IL-6 (174G/C) and TGF-β1 (509C/T) may not be associated with OLP risk in our population.

**Conclusion:**

It is concluded that IFN-γ (874A/T) polymorphism is associated with the susceptibility of OLP, however further studies with large sample size involving different ethnic populations should be conducted to strengthen our results.

## Background

Oral lichen planus (OLP) is a premalignant mucocutaneous disease in which genetic factors and immune responses play a major role. It is a chronic inflammatory, prototype of oral lichenoid lesions characterized by T-cell mediated immune response and abnormal epithelial keratinization cycle in which Th1 is considered to play a predominant role [1–4]. The OLP lesions may coexist with cutaneous and genital lesions, or may be the only disease manifestations. The OLP with complex pathogenesis, involves antigen presentation by the oral keratinocytes either of an exogenous or an endogenous origin [5]. This immune response is accompanied by a mixed inflammatory response of T-cells, macrophages, and mast cells, together with the associated cytokines and cytotoxic molecules [5, 6]. OLP is more common in middle aged women [7]. The genetic factors influencing immune function have been indicated to contribute to the OLP etiology [4, 8]. Cytokines play an important role in the progression/ pathogenesis of OLP and polymorphisms in cytokines genes such as IFN-γ, TNF-α, TNF-β, IL-4, IL-10 have been associated with the susceptibility of OLP [9, 10].

Interferon gamma (IFN-γ), a proinflammatory cytokine has also been reported to play an important role in host defense and immune regulation. The gene encoding IFN-γ is located on chromosome 12q24 and consists of four exons with three intervening regions [11]. A polymorphism in the first intron of IFN-γ gene at position 874 (rs2430561) directly influences IFN-γ production level [12]. The IFN-γ (874 A/T) is located within a putative nuclear factor-kB (NF-kB) binding site, and T allele might be responsible for the induction of IFN-γ production at a higher level.

Interleukin -6 (IL-6) is one of the major inflammation related cytokines [13]. It is responsible for the synthesis of acute-phase reactants by the liver and regulates inflammatory/ immune pathways, bone metabolism and endocrine functions. The human IL-6 gene is located on chromosome 7p21. Among the polymorphic sites described in the IL-6 gene promoter, two biallelic polymorphisms, 572G/C (rs1800796) and −174G/C (rs1800795) have been associated with differences in cytokine production. These polymorphisms consist of a single nucleotide change from guanine (G) to cytosine (C) at positions −572 and −174 in the promoter region, respectively [14, 15]. Some researchers have investigated a possible association between IL-6 (−174G/C) polymorphism and OLP in Taiwanese and Brazilian patients [16, 17].

Another cytokine, transforming growth factor (TGF)-β1 is produced by both immune and non-immune cells and exhibits a broad range of functions including tissue repair and immune response [18]. The TGF-β1 gene is located on chromosome 19q13 [19] and its production is under genetic control [20]. TGF-β1 gene polymorphisms at codon 10 and 25 regulate the TGF-β1 production in vivo and in vitro [21]. The most thoroughly studied TGF-β1 (509C/T) polymorphism (rs1800469) is located within a Yin-Yang1 consensus binding site [22] and T allele has been associated with increased level of TGF-β1 in plasma [20] and reduced T cell proliferation [21].

The cytokines involved in inflammation- and immune regulation has been suggested to play an important role in the pathogenesis of OLP. The genotype frequencies of polymorphisms are known to vary according to race or ethnicity. To date, no studies have been performed in Saudi Arabian patients to evaluate whether the IL-6, IFN-γ and TGF- β1 gene polymorphisms are associated with OLP susceptibility. We evaluated the association of IFN-γ (874A/T), IL-6 (174G/C) and TGF-β1 (509C/T) polymorphisms with OLP risk in Saudi patients.

## Methods

Two hundred thirty seven Saudi subjects visiting Prince Sultan Military Medical City (PSMMC), Riyadh Saudi Arabia were recruited for this study. Forty two unrelated OLP patients (16 male, 26 female) aged 27–72 years and 195 unrelated healthy matched controls from the same ethnicity (100 male, 95 female) aged 20–65 years were genotyped for polymorphisms in IFN-γ and TGF- β1 and IL-6 genes. Patients and controls with the history of any other inflammatory/ autoimmune diseases were excluded from the study. The study protocol was approved by the research and ethical committee of PSMMC, Riyadh and written informed consent was obtained from each subject before recruitment.

The diagnosis of OLP was based on the clinical manifestations and histopathological criteria of the World Health Organization. Senior oral pathologists examined the patients, reviewed histological findings and the patients history to diagnose OLP as described elsewhere [9]. The patient suspected to have drug or restoration related lichenoid lesions, and with any histologic signs of dysplasia were excluded.

### PCR amplification

Genomic DNA was extracted from the peripheral blood samples of OLP patients and controls using QIAamp^R^ DNA mini kit (Qiagen Hilden, Germany). IFN-γ gene was amplified using amplification refractory mutation systems (ARMS)-PCR methodology to detect polymorphisms at position 874 of IFN-γ. DNA was amplified in two different PCRs with a generic antisense primer and one of the two allele specific sense primers (antisense: 5’- TCA ACA AAG CTG ATA CTC CA-3’ T- allele: 5’- TTC TTA CAA CAC AAA ATC AAA TCT-3’ and A-allele: 5’- TTC TTA CAA CAC AAA ATC AAA TCA-3’). For quality control and to check the success of PCR amplification in both the reactions, an internal control of 426 bp was amplified using a pair of primers for the human growth hormone (HGH). PCR amplification was carried out using 5× FIREPol Master Mix (Solis Biodyne, Tartu, Estonia) with specific optimized reaction conditions. The amplified products were separated on the 1.5 % agarose gel, stained with ethidium bromide and photographed.

IL-6 (174 G/C) polymorphism was detected by PCR-RFLP technique using a set of forward and reverse primers [23]. Amplification of genomic DNA using specific protocol yielded a198 bp DNA. The PCR product (198 bp) DNA was digested with SfaNI restriction enzyme (New England BioLabs, Beverly, MA) at 37 ° C for 3 h. Resulting into two fragment of 140 and 58 bp indicating GG genotype, while three fragment of 198, 140 and 58 bp indicating GC genotype. Undigested single band of 198 bp indicated CC genotype. The amplified products and digested products for various samples were separated on the 2.5 % agarose gel, stained with ethidium bromide and photographed.

Genotyping for TGF-β1 (509C/T) polymorphisms was performed using PCR-RFLP technique. Amplification of a TGF-β1 using specific primers was performed to get 441 bp PCR product which was digested with restriction enzyme Bsu36 I to get two fragments of 251 and 190 bp for CC, three fragments of 441,251 and 190 bp for CT and uncut DNA of 441 bp for TT genotype. The alleles and genotypes frequencies were calculated in patients and control groups. Hardy-Weinberg equilibrium was determined as described earlier [9].

### Statistical analysis

The differences in allele/genotype frequencies between patient and control groups were analyzed by the Fisher’s exact test using the CalcFisher software (www.jstatsoft.org/article/view/V008i21/Article-JSS). *P* values ≤ 0.05 were considered significant. The odd ratio interpreted as *relative risk* (RR) was calculated following the Woolf’s method as out lined by Schallreuter et al. [24]. Etiologic fraction (EF) indicating the hypothetical genetic component of the disease and preventive fraction (PF) showing the hypothetical protective effect of one specific allele/ genotype for the disease were calculated using formulas given by Svejgaard [25].

## Results

**Fig. 1:**
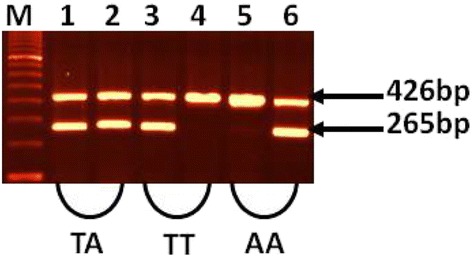
Shows the amplification of IFN-γ (874A/T) genotypes (TT, TA and AA). Lane M: 100 bp DNA marker, Lane 1 and 3: amplification of allele T, Lane 2 and 6: amplification of allele A (taking both alleles together: lanes 1 and 2 indicate TA genotype, lanes 3 and 4, TT genotype and lanes 5 and 6 AA genotype), 265 bp band for target DNA, 426 bp band for internal control

**Fig. 2:**
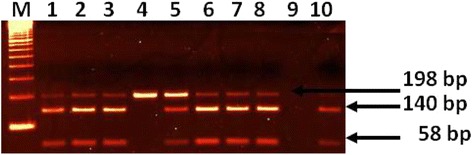
Amplified DNA digested with SfaNI showing genotypes of IL-6(174G/C). Lane M: 100 bp DNA marker, Lane 1, 2, 3,5,6,7 and 8 for genotype GC (3 bands of 198, 140 and 58 bp), Lane 4 for genotype CC (uncut DNA of 198 bp), Lane 10 for genotype GG (2 bands of 140 and 58 bp)

**Fig. 3:**
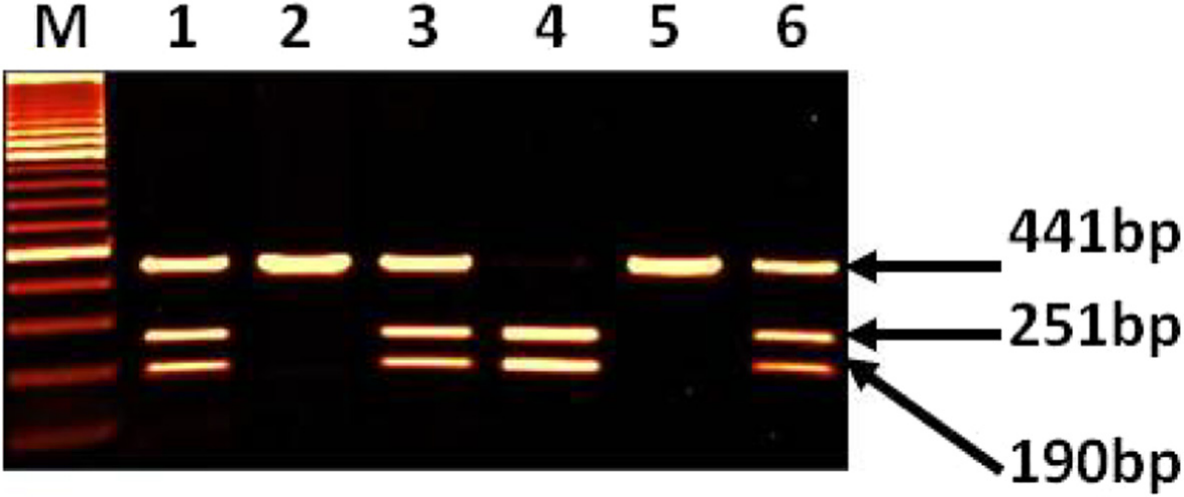
Amplified DNA digested with Bsu36 I showing genotypes of TGF-β1 (509C/T). Lane M: 100 bp DNA marker, Lane 1, 3 and 6 for genotype CT(3 bands of 441, 251and190bp), Lane 2 and 5 for genotype TT (uncut DNA of 441 bp), Lane 4 for genotype CC (2 bands of 251 and 190 bp)

The genotype and allele frequencies of IFN-γ (874A/T), IL-6(174G/C) and TGF-β1 (509C/T) polymorphisms are presented in (Tables 1, 2 and 3). The representative gel pictures of amplification or after restriction enzyme digestion for IFN-γ, IL-6 and TGF-β1 are shown in Figs. 1, 2 and 3. The results of the genotyping repeated for 30 % of the random blind sample were compared with 100 % success rate. The genotype distributions were in Hardy-Weinberg equilibrium in both, OLP patient and control groups.

**Table 1:** Genotype and allele frequencies of IFN-γ (874A/T) polymorphism in OLP patients and matched controls

Genotypes/Allele	OLP (*N* = 42)		Control (*N* = 195)		*P*- value	*RR*	*EF* ^b^/*PF*
	*N*	%	*N*	%			
AA	2	4.76	49	25.13	0.003^a^	0.149	0.182
AT	27	64.29	91	46.66	0.04^a^	2.057	0.117^b^
TT	13	30.95	55	28.21	0.71	1.141	0.023^b^
AT + TT	40	95.24	146	74.87	0.003^a^	6.712	0.183^b^
A-allele	31	36.90	189	48.46	0.07	0.622	0.070
T-allele	53	63.10	201	51.54	0.07	1.607	0.078^b^

*N* number of subjects, *RR* relative risk, *EF* = etiological fraction, *PF* preventive fraction

^a^statistically significant

^b^data for EF

**Table 2:** Allele and genotype frequencies of IL-6 (174G/C) polymorphism in oral lichen planus and controls

Genotype/Allele	OLP (*N* = 42)		Control (*N* = 195)		*P*-value
	*N*	%	*N*	%	
GG	0	0	1	0.51	0.99
GC	42	100	193	98.98	0.99
CC	0	0	1	0.51	0.99
G-allele	42	50	195	50	0.99
C-allele	42	50	195	50	0.99

*N* number of subjects

**Table 3:** Allele and genotype frequencies of TGF-β1 (509C/T) polymorphism in OLP and controls

Genotype/allele	OLP (*N* = 42)		Control (*N* = 195)		*P*-value
	*N*	%	*N*	%	
CC	11	26.19	40	20.51	0.41
CT	27	64.29	115	58.98	0.60
TT	4	9.52	40	20.51	0.12
C-containing	38	90.48	155	79.49	0.12
C-allele	49	58.33	195	50	0.18
T-allele	35	41.67	195	50	0.18

*N* number of subjects

The frequencies of genotypes and alleles of IFN-γ (874A/T) polymorphism differed in OLP patients and controls (Table 1). The frequency of heterozygous genotype (AT) of IFN-γ was significantly higher in OLP patients than control (*P* = 0.04) whereas the frequency of homozygous genotypes (AA) was significantly lower in OLP patients than controls (*P* = 0.003). The frequency of T-containing genotypes (AT + TT) were significantly higher in OLP patients as compared to that in controls [*P* = 0.003 and relative risk (RR) = 6.712]. The frequency of allele T was higher and that of allele-A was lower in OLP patients than control subjects however, the difference was slightly short of significant (*P* = 0.07). On the other hand the difference in frequency of TT genotype among the two groups was not statistically significant (*P* = 0.71).

The genotype and allele frequencies of IL-6 (174G/C) polymorphism did not differ between patients and controls (Table 2). Both the homozygous genotypes CC and GG were absent in OLP patients. The heterozygous genotype GC was present in all OLP patients while 98.98 % of the controls had this genotype. The frequencies of allele G and C were exactly similar in controls and OLP patients.

The distribution of frequencies of alleles and genotype of TGF-β1 (509C/T) polymorphism did not differ significantly between OLP patients and healthy controls (Table 3). The frequencies of genotypes CC, CT and AA were 26.19, 64.29 and 9.52 % in OLP patients as compared to 20.51, 58.98 and 20.51 in controls respectively. The distribution of allele C and T was quite similar, and it was respectively 58.33 and 41.67 % in OLP patient group and 50 % each in healthy control group (*P* > 0.05).

The frequency distribution of genotypes of IFN-γ (874A/T) polymorphism in various healthy ethnic populations is summarized in Table 4 which clearly indicated ethnic variations.

**Table 4:** Genotypes frequencies of IFN-γ (874A/T) polymorphism in different healthy populations

Population	Subjects number	Genotypes			References
		AA	AT	TT	
Saudis	195	49 (25.13)	91 (46.66)	55 (28.21)	Present study
Brazilian	186	64 (34.41)	77 (41.40)	45 (24.19)	[46]
Brazilian	76	17 (22.37)	46 (60.53)	13 (17.10)	[47]
Brazilian	191	60 (31.41)	34 (17.80)	97 (50.79)	[48]
Canadian	91	27 (29.67)	41 (45.06)	23 (25.27)	[49]
Chinese	480	212 (44.17)	201 (41.87)	67 (13.96)	[50]
Chinese	97	77 (79.38)	17 (17.53)	3 (3.09)	[51]
Chinese	128	101 (78.91)	26 (20.31)	1 (0.78)	[52]
Egyptian	118	6 (5.08)	60 (50.85)	52 (44.07)	[53]
Egyptian	106	8 (7.5)	92 (86.8)	6 (5.7)	[54]
Finnish	63	23 (36.51)	31 (49.21)	9 (14.28)	[55]
German	561	9 (1.60)	136 (24.24)	416 (74.15)	[56]
Greek	39	5 (12.82)	23 (58.97)	11 (28.21)	[57]
Indian	881	281 (31.90)	435 (49.37)	165 (18.73)	[58]
Indian	374	90 (24.07)	178 (47.59)	106 (28.34)	[59]
Indian	150	66 (44)	63 (42)	21 (14)	[60]
Iranian	354	123 (34.75)	134 (37.85)	97 (27.40)	[61]
Iranian	539	138 (25.60)	248 (46.01)	153 (28.39)	[62]
Israeli	48	18 (37.5)	24 (50)	6 (12.5)	[63]
Italian	140	42 (30)	66 (47.14)	32 (22.86)	[26]
Italian	96	30 (31.25)	51 (53.13)	15 (15.62)	[64]
Japanese	188	157 (83.51)	31 (16.49)	0 (0)	[65]
Korean	201	151 (75.12)	47 (23.38)	3 (1.50)	[66]
Polish	74	10 (13.51)	49 (66.22)	15 (20.27)	[67]
Thai	154	92 (59.74)	53 (34.42)	9 (5.84)	[68]
Thai	137	83 (60.58)	46 (33.58)	8 (5.84)	[69]
Tunisian	113	33 (29.20)	47 (41.60)	33 (29.20)	[70]
Turkish	40	15 (37.5)	21 (52.5)	4 (10)	[71]
Turkish	71	31 (43.66)	31 (43.66)	9 (12.68)	[72]
Turkish	99	16 (16.16)	56 (56.57)	27 (27.27)	[73]

## Discussion

Genotyping results of IFN- γ (874A/T) polymorphism indicated that T-containing genotypes are significantly associated with the susceptibility to OLP in Saudis (*RR* = 6.712). The IFN-γ is an important predominant cytokine in the pathogenesis of OLP. The role of IFN-γ gene polymorphism on OLP susceptibility has been investigated in various populations. Studies from China, Thailand and Italy indicted an association between IFN-γ (874A/T) polymorphism and OLP [26–28] and it has been suggested that IFN-γ (874A/T) polymorphism may be a risk factor to OLP development.

Carrozzo et al. [26] found significant increase in number of TT homozygous genotype of IFN-γ (874A/T) polymorphism in OLP patients compared with controls and suggested that IFN-γ genetic polymorphism may be an important risk factor to develop OLP in Italian. Bai et al. [27] suggested that IFN-γ (874A/T) polymorphism is associated with susceptibility and influence progression of OLP in a Chinese cohort. Recently Kimkong et al. [28] also reported an association between IFN-γ (874A/T) polymorphism and susceptibility to OLP in Thai population and suggested that the T allele is significantly associated with an increased risk of OLP development as compared to the A allele. Several other studies have also shown that IFN-γ +874 T/A polymorphism is associated with the development of several autoimmune diseases while others showed no such associations [29]. These disparities are probably caused by small sample sizes, low statistical power, ethnic difference, and/or clinical heterogeneity as suggested by Lee and Bae [29].

The results of present study on the associations of the IFN-γ 874 T/A polymorphism with OLP are consistent with the functional effect of the IFN-γ 874 T/A polymorphism as genotypes of the IFN-γ 874 T/A polymorphism have been associated with low (AA), medium (AT), and high (TT) cytokine production [12]. The IFN-γ 874 T/A polymorphism has been associated with autoimmune diseases in Caucasian, Latin American, and Middle Eastern, but not Asian, populations as shown in a recent meta-analysis [29]. The reason why a particular polymorphism was found to be associated with one population and not another may be due to ethnic differences in the frequency of IFN-γ 874 T/A polymorphism among these ethnic groups. The frequency distribution of genotypes of IFN-γ (874A/T) polymorphism in various healthy ethnic populations clearly indicated ethnic variations (Table 4). The frequency of AA genotype varies from 1.60 % in German to 83.51 % in Japanese while frequency of AT genotype from 16.49 % in Japanese to 66.22 % in Polish population. On the other hand the frequency of TT genotype varies from 0.78 % in Chinese to 74.15 % in German healthy population however it was totally absent in Japanese healthy population. Obviously, there is a difference in the frequency of the T allele of the polymorphism among the healthy ethnic groups. The T allele was a minor allele in Asian populations, whereas it was a major allele in Caucasian and Latin American populations [29]. Our previous study dealing with the polymorphisms of pro- and anti-inflammatory cytokines showed that the OLP patients has significantly higher frequency of the A allele of TNF-α (−308) polymorphism [9]. Notably, both the polymorphisms (IFN-γ and TNF-α are known to increase the production of the respective cytokine [30, 31]. Significantly, several studies demonstrate a high level of IFN-γ and TNF-α in the oral mucosa [32] and serum of the patients with OLP [33].

INF-γ is one of the most critical mediators of immunity and inflammation and plays a pivotal role in both innate and adaptive immune responses [34]. It has been shown to promote innate immune responses by activating macrophages. Further by disrupting several anti-inflammatory feedback loops, INF-γ also up-regulates various proinflammatory mediators [35]. IFN-γ is known to enhance Th1 responses by activating NK cells and macrophages. It also promotes the specific cytotoxic immunity via T cell and APC interaction [36].

Moreover, IFN-γ is among the most extensively studied cytokines in OLP. Higher expression of IFN-γ has been reported in the isolated T-cell lines from the OLP biopsies [2, 37] and in erosive OLP lesions [3, 37]. Its expressions have been located on the CD4 + Th cells in OLP lesions [38]. Sugerman et al. [1] suggested that the high expression of IFN-γ at the advanced stage of OLP development may be involved in the activation of CD8 + T cells and help in maintaining the expression of major histocompatibility class on the keratinocyte. The increased expressions of IFN-γ in OLP influence the clinical outcome and has been associated with the clinical manifestations of OLP lesions [39].

On the other hand, in this study no significant difference in the allele or genotype frequencies of IL-6 (174G/C) were observed between the OLP subjects and healthy controls (Table 2). In contrast to our results some reports have indicated an association of IL-6 (−174 (G/C) with the OLP susceptibility. Xavier et al. [17] found a significant higher frequency of IL-6 174 G/G genotype in a cohort of Brazilian patients with OLP and suggested an association with the susceptibility of OLP and involvement of this polymorphism in the genetic basis of this disease.

Recently the IL-6 (174G/C) polymorphism has also been significantly associated with oral precancerous lesions (OPCLs) in Taiwanese patients [16]. These differences in the results can be attributed to the ethnic variations in the distribution of polymorphic variants as the genotypes/alleles frequencies of polymorphisms are known to vary according to race or ethnicity.

Further, we did not notice any significant difference in the allele or genotype frequencies of TGF-β1 polymorphism between the OLP patients and healthy controls in this study. Frequency of the T allele of TGF- β 1 (509 C/T) polymorphism in the OLP patient group was 41.67 % as compared to 50.0 % in the control group. Genotypes (TT, TC, and CC) frequencies of TGF-β 1 (509 C/T) polymorphism among the patients and the healthy controls though differed but the difference was not statistically significant (Table 3). In accordance with our results, a study from Italy also repoted no significant difference in the allele or genotype frequencies of TGF-β1 between the OLP subjects and healthy controls [26]. Recently Hsu et al. [16] suggested that polymorphism in TGF-β 1 (C/T) (rs1800469) is not associated with OPCLs but another polymorphism TGF-β 1 (C/G) at codon 25 is associated significantly with the development of OPCLs in Taiwanese.

TGF-β plays highly significant role in the immune system. It regulates the IFN-γ production by NK cells. It can help accumulate the pro-inflammatory macrophage (M1) to the anti-inflammatory type (M2) [40]. On the other hand inhibition of TGF-β pathway in the lymphocytes contributes to the chronic inflammation in OLP lesions [41] which is partly attributed to the over productions of IFN-γ, leading to the blockage of the phosphorylation of Smad3 [41]. The balance between TGF-β and IFN-γ signaling determines the immunological status and can be a therapeutic target in OLP patients [42]. The available literature on *TGF*-*β* 1 polymorphism is inconsistent, as some reports support the fact that the polymorphism is associated with susceptibility to the disease while others suggest that it may be protective [43–45].

## Conclusion

It is concluded that the IFN-γ (874A/T) polymorphism is associated with the susceptibility to OLP. On the other hand the polymorphisms IL-6 (174G/C) and TGF-β1 (509C/T) may not be associated with OLP risk in our population, however further studies with large sample size involving different ethnic populations should be conducted to strengthen our results.

## Abbreviations

EF: Etiological fraction
IFN-γ: Interferon gamma
IL-6: Interleukin-6
OLP: Oral lichen planus
OPCLs: Oral precancerous cells
PCR-RFLP: Polymerase chain reaction -restriction fragment length polymorphism
PF: Preventive fraction
RR: Relative risk
TGF- β: Transforming growth factor beta
